# Implications of the Circular Economy in the Context of Plastic Recycling: The Case Study of Opaque PET

**DOI:** 10.3390/polym14214639

**Published:** 2022-10-31

**Authors:** Noel León Albiter, Orlando Santana Pérez, Magali Klotz, Kishore Ganesan, Félix Carrasco, Sylvie Dagréou, Maria Lluïsa Maspoch, César Valderrama

**Affiliations:** 1 Centre Català del Plàstic (CCP)—Universitat Politècnica de Catalunya Barcelona Tech (EEBE-UPC), ePLASCOM, Avda, Eduard Maristany, 14, 08019 Barcelona, Spain; 2 Chemical Engineering Department, UPC-BarcelonaTECH, C/Eduard Maristany, 10-14 (Campus Diagonal-Besòs), 08019 Barcelona, Spain; 3 Barcelona Research Center for Multiscale Science and Engineering, C/Eduard Maristany, 10-14 (Campus Diagonal-Besòs), 08019 Barcelona, Spain; 4 Department of Chemical Engineering, Universitat de Girona (UdG), C/Maria Aurèlia Capmany 61, 17003 Girona, Spain; 5 CNRS, Institut Des Sciences Analytiques et de Physico-Chimie Pour l’Environnement et les Matériaux (IPREM), Université de Pau et des Pays de l’Adour, E2S UPPA, UMR5254, 64053 Pau, France

**Keywords:** circular economy, upcycling, sustainability, polyblends, rPP, PA66, rPET-O

## Abstract

The use of recycled opaque PET (r-O-PET, with TiO_2_) as a reinforcement for the recycled polypropylene matrix (r-PP) was evaluated through the life cycle assessment according to different scenarios corresponding to two different recycled blends and considered two virgin raw plastic material as reference materials when comparing the environmental performance of the proposed treatments. The results indicate that the environmental performance was quite different for each blend, since the additional extrusion process required in scenario 2 (blend with TiO_2_) causes all impact categories analysed to report higher values when compared with scenario 1 (blend without TiO_2_). The stage that contributes the most corresponds to the different extrusion processes included in both recycling blends, representing at least 80% of the total for global warming. Compared with virgin raw materials, the blend with TiO_2_ showed better performance in all the impact categories analysed in comparison with virgin PA66, while the blend without TiO_2_ showed the opposite trend when compared to PP. Furthermore, the fact that the upcycling treatment was carried out on a pilot scale provides room for improvement when implemented on a full scale. It is worth noting the high energy consumption of the treatment processes and their associated cost, in addition to the market cost of virgin raw materials, however, when considering the environmental cost of raw materials, it is observed that when substituting virgin materials PP and PA66 for the blends evaluated in this study results in a reduction of the environmental price of up to 2.5 times.

## 1. Introduction

In the past decade, global awareness regarding environmental issues (global warming, climate change, resource depletion…) has increased considerably. Plastic pollution is of particular concern, given that due to their numerous benefits, plastics have become ubiquitous throughout society and, consequently the amount of post-consumer plastic waste is increasing, leading to significant environmental drawbacks. In the absence of action, the amount of plastic waste produced globally is predicted to triple between 2015 and 2060, to between 155 and 265 million tonnes per year [1].

Governments and policymakers have started to understand the need to transition to more sustainable systems. Hence, in 2011 European Union designated resource efficiency as one of the flagships of its Europe 2020 Strategy (turning wastes into a resource) [2] and the EU settled in July 2014 the target of recycling at least 80% of plastic packaging waste by 2030 and banning burying recyclable waste in landfill as of 2025 [3]. In January 2018, Plastics Europe released its ‘2030 Plastic Voluntary Commitments’, announcing its willingness to achieve the 100% re-use, recycling and/or recovery target of all plastics packaging in the EU-28, Norway and Switzerland by 2040 [4].

In this context, the circular economy (CE) approach promotes more effective use of materials by creating more value, for instance, promoting the cycle of high-value material instead of recycling only for law value raw materials as in traditional recycling [5,6]. Through various principled actions, it is possible to operate a closed-loop ecosystem and extend the life-cycle of products, equipment and infrastructure. Thus, improving the resource utilization, reducing waste, and energy consumption.

Regarding plastic packaging, the Ellen MacArthur Foundation’s report [7] concreted a set of three priority actions to trigger the transition towards a new economy: (i) the fundamental redesign of 30% of plastic packaging that otherwise will never be reused or recycled; (ii) the reuse of at least 20% of plastic packaging; and (iii) the recycling of the remaining 50% of plastic packaging, radically improving their quality and economic attractiveness.

Despite the numerous solutions included in the CE approach, particular emphasis is still placed on the recycling of plastic packaging wastes in response to the EU targets, as well as the communities’ aspirations towards “zero waste” cities. Nevertheless, the main technical difficulties of recycling are the variability of composition and the level of contamination of the waste stream.

Recycling of plastics involves many processes including collecting the wastes from the point of production or disposal, sorting, compressing, crushing and pelletizing them into raw materials. These procedures are followed by their thermal, chemical or mechanical processing to the final product. For this reason, the recycling of plastic waste is intricate and less preferred compared to other materials such as aluminium, glass, ceramics and paper [8]. Through shredding and grinding, plastic wastes are degraded during mechanical recycling [9]. The method is however not preferable if the mixture of wastes is complex and instead, incineration is preferred [10].

Recycled plastic can be reused in a closed loop material flow, reused in less critical applications (down-cycling), or upgraded for another use (up-cycling). The upgrading strategy generally comprises several elements of the polymer blend technology, viz alloying (i.e., compatibilization and/or impact modification), blending to the desired morphology, and compounding with other additives (e.g., stabilizers and fillers) [11].

Plastic milk bottles were traditionally made of high-density polyethylene (HDPE), but in order to achieve cost savings, the use of PET started to develop in the 2010s. In addition to the fact that it is cheaper than HDPE, the use of PET reduces the weight of the container by 25% and eliminates the aluminium seal on the bottle [12,13]. It also reduces water consumption by 20% and the energy consumption of the manufacturing process by 13%. Since PET bottles are normally transparent, between 10 to 20% *w*/*w* of TiO_2_ is used as opacifying agent, conferring a screening effect from UV radiation of the content, minimising gas permeation, and a glossy white aspect when no other pigments are used.

The counterpart of opaque PET is its difficulty in being recycled by conventional processes used for transparent PET (r-T-PET), especially the bottle-to-fibre recycling process, which represents around 44% of its market share [14]. The French packaging compliance organisation Eco-Emballages stated that above a threshold of 15% by weight, the presence of recycled opaque PET (r-O-PET) in the PET bottle-to-fibre recycling stream leads to a series of difficulties during the filament manufacturing and significant deterioration of the mechanical properties of the fibres [13].

The presence of at least 0.35% *w*/*w* of TiO_2_ is capable of retard strain hardening and accompanying stress-induced crystallization, when stretched in the rubbery state (temperatures between T_g_ and T_m_), generating low levels of crystallinity and orientation. Apparently, the submicron TiO_2_ particles interfere in the formation of the physical network necessary to promote the necessary hardening in the stabilization of the stretching stage during the manufacture of the fibre. Concentrations higher than 4% *w*/*w* generate a structure with poor mechanical properties [15]. That is why, as an alternative solution, recycling companies have chosen to market a grade of r-O-PET “diluted” with r-T-PET such that the TiO_2_ content is around 2% *w*/*w*, which allows the manufacturing fibres with “acceptable” mechanical properties.

Taking into account that in 2016 opaque PET already represented an average of 12% by weight of the materials managed in the bottle-to-fibre recycling streams in France and that the market continues to grow. Therefore, if no action is taken recyclers will have no choice but to send opaque PET bottles to landfill or incineration, as the treatment systems cannot tolerate such quantities. Consequently, this waste stream will have a significant environmental impact and therefore will not follow the European strategies for plastics within the circular economy policy [16].

To address this growing issue, Eco-Emballages launched an action plan in 2017 [13], divided into three actions. One of these actions aims to search for an added-value market for r-O-PET. The case study presented in this work is framed within this context. The rationale behind the idea was to recover the opaque PET waste stream and improve its mechanical/physical properties and consequently its economic value while reducing its environmental impacts, which is in line with the CE approach.

In a previous study, it was observed that the presence of almost 2% *w*/*w* of TiO_2_ on r-T-PET promotes an increase of 21% in the energy required for crack propagation. This effect is caused by the TiO_2_ sub-micron particle cavitation, which allows a significant release of local triaxiality, promoting plastic the premature plastic tearing of the PET matrix [17]. Thus, one of the upgrading pathways proposed considered in this case study was to explore the use of r-O-PET as a reinforcement for recycled polypropylene (r-PP) matrix, inducing a microfibrillation of the PET phase during processing that acts as reinforcing fibres, under the manufacturing philosophy of in situ microfibrilated composites (MFC) [18]. According to his study, the blend composition that offers the best balance of mechanical properties and fracture behaviour of the MCM produced is the one with 20% *w*/*w* of r-O-PET. However, due to the intrinsic immiscibility of both polymeric phases, and considering the relatively low amount of TiO_2_ compared to the actual content in the bottles, the inclusion of superficially modified TiO_2_ in the blend was tested in order to evaluate its effectiveness to enhance the compatibility between the two polymeric phases. According to the results, the use of an hydrophobic treated particle until 12% *w*/*w* induces an emulsifying effect of the r-O-PET phase and good compatibilization in the elastic regime of mechanical performance [19].

The objective of this study is to evaluate two different solutions for the opaque PET waste stream, two different blends of r-O-PET have been proposed and produced on a pilot scale and then tested on an industrial scale to produce components for the electrical industry. In order to evaluate the environmental performance of the two blends, the life cycle assessment (LCA) methodology is used and, in addition, it is compared with virgin plastic materials of fossil origin that are commonly used to manufacture the same components. The aim is to evaluate the environmental cost over the life cycle of recycling the opaque PET stream and that of competing for virgin materials for the same application. The objective is also to illustrate the challenges that users/supporters of the CE approach must overcome when it comes to recycling plastic packaging, especially in terms of promoting high-added-value applications and creating new markets.

## 2. Materials and Methods

### 2.1. Materials and Scenarios

It is worth mentioning that this study is part of a project carried out in conjunction with the Institut Des Sciences Analytiques et de Physico-Chimie Pour l’Environnement et les Matériaux (IPREM) (Pau, France). The recycled raw material for the manufacture of the proposed blends corresponds to grades marketed by Suez RV Plastiques Atlantique (Bayonne, France): flakes of a mixture of recycled O-PET mixed with recycled transparent PET to reach a nominal global content of 2% *w*/*w* TiO_2_ (r-O-PET); and a recycled PP (r-PP) from the automotive industry. Both were sent from IPREM (Pau, France) to our facilities (Spain). However, for the life cycle inventory, the original origin of both (France and The Netherlands, respectively) was considered.

The composition of the blend was selected after a preliminary study carried out on a laboratory scale. In this study, it was established that the addition of 20% by weight of r-O-PET to r-PP offered the best balance of mechanical properties. The generation of a microfibrillated morphology of the PET phase during processing promotes a reinforcement in stiffness to the system. For this composition, a global amount of TiO_2_ determined was 0.2% *w*/*w*. Additionally, and considering the possibility of increasing this TiO_2_ content, a mixture with a global content of 4% *w*/*w* of TiO_2_ was evaluated to compare its performance. Details of this study could be found in [18,19].

An industrial partner, specialising in the manufacture of injection moulded parts for different markets, carried out a study on possible parts that could be considered based on processability and mechanical performance. Two components used in the building construction industry were selected for a comparative study between the traditional petroleum-based plastic and the proposed recycled blends. The traditional virgin raw material employed in their manufacture is a Polypropylene heterophasic copolymer, ISPLEN 140 G2M (Repsol, Madrid, Spain) and a Polyamide 66, Zytel 101L BKB080 (Dupont, Wilmington, DE, USA).

Table 1 collects the values of the mechanical, physical and flow properties that were used for the selection of the comparison scenarios of this study, after the blends preparation in the pilot plant scale. It is important to note that the values reported for virgin materials (PP and PA66) correspond to the technical specifications provided by the raw material producers, while those of the blends were determined under the same conditions and following the same standards used for virgin PP.

When selecting the scenarios for the present LCA analysis, an attempt was made to prioritize similarity in terms of:

(a) *Similarity of Specific elastic modulus (Esp) values:* Ratio between elastic modulus (E) and material density (*ρ*) which is a mechanical parameter used in structural design.
(b) *Similarity in the fluidity of the melt under normal processing conditions*. In the case of the parts made from virgin PP, the proximity between MFI was used as a criterion. In the case of PA66, as this parameter is not available, this comparison is meaningless. However, the conditions for the processing of both blends (similar to a PP) offer fewer drawbacks than those used for processing a typical PA66: there is no need for drying, there is no need to use screws with anti-return and anti-drip valves, and the temperature profile is more venébolo (230 °C, for PP vs. 290 °C for PA66).

In this way, the following study scenarios were selected:

***Scenario 1:*** 20w% r-O-PET/80w% rPP vs. PP (Isplen)

***Scenario 2:*** 20w% r-O-PET/76w% rPP/4% TiO_2_ vs. PA66 (Zytel)

### 2.2. Blends Preparation at Pilot Plant Scale

The heterogeneity in the composition and geometry (flakes) of the received r-O-PET hinders any continuous forming process, which is why as the first step in blend preparation a homogenization of raw material by extrusion was performed. It used a KNETER 25 × 24D co-rotating twin screw extruder from COLLIN (Ebersberg, Germany) with a length-to-diameter ratio (L/D) of 36, a screw diameter (D) of 25 mm, and 7 heating zones (1 for die). A filament-type die of 3 mm nominal diameter was used to produce pellets after cooling in a water bathtub (1500 mm in length) at 20 °C. The processing conditions used were:

***Temperature profile (°C):*** 180/220/240/245/245/250/250 °C (die)

***Screws rotation speed:*** 55 rpm.

Under these processing conditions, the decrease in the Intrinsic Viscosity (IV) of the product was determined to be less than 10% [20].

The selected blend composition (r-PP/r-O-PET: 80/20) was prepared using the same twin screw extruder described above with the same extrusion conditions used in the homogenization step. Additionally, the same blend composition was prepared with an additional 4% by weight of TiO_2_. For this, an r-PP/TiO_2_ masterbatch was prepared and subsequently diluted with the required amounts of r-PP and r-O-PET until reach the desired proportion of additional TiO_2_ in the resultant r-PP/r-O-PET-O. This methodology assures a better dispersion and distribution of the inorganic component.

The preparation of the masterbatch was carried out in an E-30/25 single screw extruder from IQAP-LAP (Barcelona, Spain) with an L/D ratio of 25, a screw diameter of 30 mm, and 4 heating zones (1 for die). A filament die diameter of 3 mm (for pelletizing) die was used. Prior to pellets cutting the filament was cooled in a water bathtube (1500 mm in length) at 20 °C. The following processing conditions:

***Temperature profile (°C):*** 140/160/185/210 (die)

***Screw rotation speed:*** 50 rpm.

It is important to quote several aspects on PET pre-conditioning before processing:

(a) Before each processing stage, PET must be dried to minimize its high tendency to hydrolytic thermos-degradation. In this case, drying was carried out in dried during 4 h at 120 °C in a PIOVAN hopper-dryer (DSN506HE, Venice, Italy) with a dew point of −40 °C, a common industrial device in PET processing. This procedure was carried out both for the homogenization of the r-O-PET flakes and for the preparation of the proposed blends.
(b) Due to the cooling conditions used during the flake homogenization stage, the r-O-PET obtained is in an amorphous state. In order to carry out its drying under the usual conditions (see point (a) above), a recrystallization process is required in order to avoid agglomeration of the pellets. Recrystallization was performed by heating the pellets in a Dry Big 2,003,740 convection oven (JP Selecta, Barcelona, Spain) at 90 °C for 4 h, taking care every 30 min to remove the pellets to avoid agglomeration in this step.
(c) To further minimize the inevitable hydrolytic thermodegradation of the PET phase during flake homogenization, an N_2_ blanket was introduced into the feeding zone.

### 2.3. LCA Methodology

#### 2.3.1. Functional Unit and System Boundaries

The functional unit is 1 kg of plastic granules produced at the gate of the treatment facility for both scenarios. It means that all the flows involved in the system were calculated or estimated in order to obtain 1 kg of the blends according to the composition listed in Table 1. The system boundaries include the processes from raw materials to plastic pellets, and the compounding with additives as well. The production of raw materials in both scenarios corresponds to the recycling stages of opaque PET and PP waste. The scope of the analysis is restricted from cradle to gate, as recycled blends and virgin materials are assumed to be equivalent for the market regarding their mechanical properties as well as their processing window.

**Figure 1:**
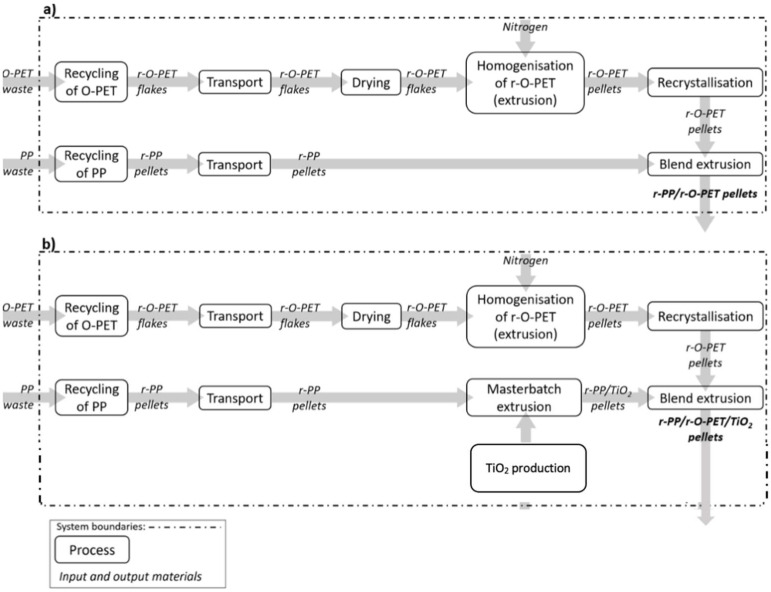
Schematic flow charts cradle-to-gate system boundaries for the recycling opaque PET waste for (**a**) Scenario 1 and (**b**) Scenario 2.

The schematic flow charts of the processes under consideration in the LCI system boundaries for both scenarios are shown in Figure 1. As is conventionally conducted in literature for open-loop recycling processes, a “cut-off” approach is applied, therefore it is not considered the first life of plastic waste [21]. The main difference between the two scenarios is the presence of an additional extrusion process to prepare the r-PP/TiO_2_ master batch in Scenario 2.

#### 2.3.2. Life Cycle Inventory

The recycling stages of opaque PET (O-PET) and PP include the first phase of plastic waste collection, transport, initial sorting and balling, as well as the second phase of plastic waste reprocessing. First, the PET waste is unbaled, pre-washed in order to remove labels, sorted by colour and chopped into flakes. The obtained flakes are then washed, rinsed and dried. On the other hand, the PP waste is washed, sorted, shredded into flakes and pelletised. Therefore, the r-O-PET flakes are produced in France and r-PP pellets are produced in the Netherlands. It is assumed that they both are transported by lorry to Spain, where the blend preparations take place in a pilot-scale extrusion plant. The transport stages included in the system boundaries are listed in Table 2.

The energy consumption during the pre-conditioning stage of r-O-PET (flakes and homogenized pellets) was estimated form the calibration curve Temperature vs. electrical power provided by the manufacturer of the Dryer hopper and convection oven.

The material loss for the extrusion processes is estimated between 7 and 10w%, due to the characteristics of the tests carried out at the pilot scale, the operation is not continuous and there are material losses every time the plant is started and during the stabilization process. The construction of plants and equipment, as well as the maintenance of plants and machinery and the end-of-life treatments of the waste generated by the blending processes, are out of the scope of the present study.

#### 2.3.3. Input Data and Assumptions

The data for r-O-PET flakes and r-PP pellets production is derived from eco-profiles made by SRP (French syndicate of manufacturers of recycled plastics) in 2017 [22].

Although the data for recycled PET is estimated from the data for transparent PET, it is assumed that the results would be similar for the production of r-O-PET flakes. This assumption comes from the fact that uncoloured transparent PET and coloured transparent PET are already recycled separately, but no distinction is made between the two in the LCI data provided by SRP.

The dataset of r-PP pellets provided by SRP is estimated for France. It is assumed in this study that data would be similar for polypropylene recycled in the Netherlands.

On-site primary data collection was performed for the processes of flake homogenization, re-crystallization, masterbatch preparation, and r-O-PET blend preparation. In these processes, energy, water and resource consumption, waste generation and the quantities of intermediate products obtained in each process were determined. These values were used to prepare the inventory expressed in terms of the functional unit (1 kg of pellets) for the two scenarios.

The Ecoinvent database was used to model transports, electricity and heat production and the production of additional substances that are required for processes. The datasets provided by Ecoinvent used in the LCIA calculations are listed in Table 3. The data for petroleum-based polymers (virgin polymers) is derived from eco-profiles performed by Plastics Europe in 2014 [23,24].

#### 2.3.4. Environmental Impact Assessment

A conventional set of impact categories is defined in the CML IA baseline 2001< (Version 4.7) (Updated in August 2016) Method and is used to assess impacts on the mid-point levels: abiotic resource depletion potentials (ADPs) for fossil fuels and elements (ADP fossil, ADP elements), climate change (GWP100), eutrophication (EP), acidification (AP) and ozone layer depletion (ODP, steady state). This baseline characterisation method as well as the aforementioned impact categories were selected to allow a direct comparison with the environmental performances of virgin polymers, which were obtained through a secondary research database (Ecoinvent v3.8).

## 3. Results

### 3.1. Life Cycle Inventory and Environmental Performance of Recycling Scenarios

The flows of materials and products involved in the production of 1 kg of plastic blend pellets for both scenarios are summarized in Table 4. the results for ADP fossil, ADP elements, GWP100, EP, AP and ODP categories obtained for both scenarios are collected in Table 5.

**Figure 2:**
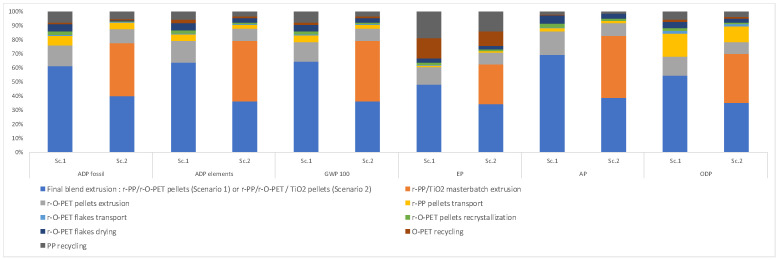
Contribution of the life cycle stages of the treatment processes to the environmental impact categories according to the CML method for both recycling scenarios.

The environmental performance of the blends is quite different for all the environmental categories summarized in Table 5, although the LCI for both is quite similar (Table 4). The additional extrusion process required in scenario 2 causes all impact categories assessed to report higher values when compared with scenario 1. This increase is significant across all impact categories. The contribution of each process involved in the treatment of the recycled blends is shown in Figure 2.

The stage that contributes the most corresponds to the different extrusion processes included in both recycling blends, representing at least 80% of the total to the global warming, the abiotic elements, abiotic fossil, acidification potential, eutrophication and ozone depletion potential; it is due to the electricity consumption in these processes. It is worth mentioning that other stages such as recrystallization, drying and transportation of the pellets reported low contributions in all the impact categories evaluated.

In the case of Scenario 1, for all impact categories, the extrusion of the final blend represents approximately 60% of the total contribution of extrusion processes, meanwhile, the extrusion of r-O-PET pellets corresponds to 15%. The high contribution of extrusion processes is due to the electricity consumption, since it corresponds to approximately 84% of the total electric energy used in the system boundaries (Table 4).

In the case of Scenario 2, which includes an additional extrusion step, the total percentage of the extrusion processes contribution is higher than the one obtained for Scenario 1. Moreover, for all impact categories, an increase between 40% and 80% is observed (Table 5). The share of contribution between the different extrusion processes is also redistributed for all impact categories. The final blend extrusion step and the r-PP/TiO2 masterbatch extrusion are the major contributors (approximately 37% of the total contribution across categories), followed by the r-O-PET homogenization step (around 9%). It clearly illustrates the effect of the added extrusion step in Scenario 2, leading to an increase in extrusion processes contribution (Figure 2) and in consequence an increase in environmental impacts (Table 5). As in the case of Scenario 1, the aforementioned observations can be linked to the electrical energy consumption, the addition of the masterbatch extrusion process leads to an increase of 44% of the total electricity consumption (Table 4).

Regarding the contributions of the O-PET and PP recycling stages, it was observed they contribute mainly to the EP impact category, with each recycling stage contributing around 16% and 12% for scenarios 1 and 2, respectively. It is worth mentioning that these stages are carried out in France and that the French electricity mix is mainly dominated by nuclear energy (>70% of the electricity grid) [20]. Nuclear power plants use cooling water that is then discharged back into the environment at a temperature typically around 30–40 °C and this thermal water pollution can lead to eutrophication [25].

### 3.2. Comparison of Blends with Raw Virgin Materials

**Figure 3:**
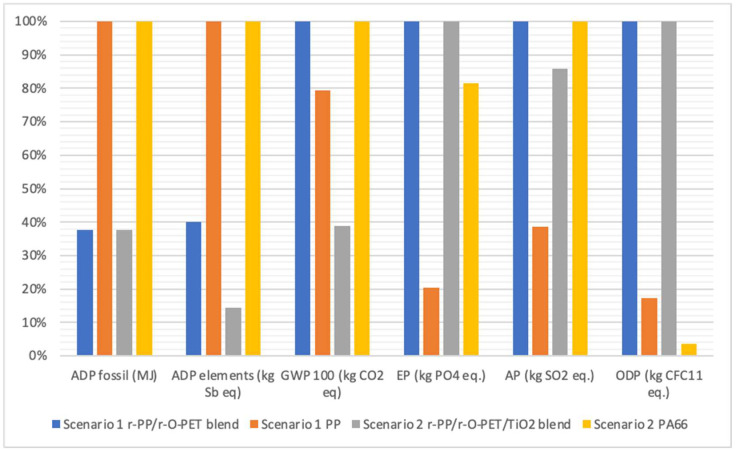
Relative indicator results of the impact categories for both scenarios. For each indicator, the maximum result is set to 100%.

It is worth noting that the two blends (scenario 1 and scenario 2) have been assumed to be secondary raw materials that can replace virgin PP and virgin PA66, respectively, which means assuming that both products have the same quality and the necessary properties that allow their use in certain applications. It is also important to highlight the fact that the results presented in this study for recycled products were obtained experimentally in a pilot plant located in a specific location, while the results of the virgin polymers correspond to the ecoinvent database. Polypropylene granulate production for Europe has been used to represent virgin PP production and nylon 6 production for Europe (derived from the Eco-profiles of the European plastics industry (PlasticsEurope)) was used to represent virgin PA 66 production. Therefore, the results presented here mainly aim to provide elements for discussion and analysis on the conceptual development of the circular economy in the context of the recycling of plastics. The comparison of both blended products and the virgin raw materials is collected in Table 6 and depicted in Figure 3.

Regarding Scenario 1, the blend without TiO_2_ reduces fossil energy depletion by more than half compared to virgin PP. The recycled blend r-PP/r-O-PET shows a higher score than virgin PP for global warming (2.38 and 1.89 kg CO_2_ eq, respectively). It can be explained, in the case of the recycled blend, by the high electricity consumption needed to produce 1 kg of plastic pellets and the low performance of the pilot plant. It is also important to stress the fact that data from the Spanish electricity grid were used for the recycled blend, meanwhile, a European mix was used for the virgin PP, as was reported by ecoinvent 3.8. The same trend is observed in the case of AP, EP, and ODP since the extrusion processes are the ones that contribute to these environmental impacts (Figure 2). Regarding the virgin PP, for all impact categories, the monomer production (i.e., crude oil, natural gas extraction and transport, the refinery, and the steam cracking and fluid catalytic cracking processes) contributes more than 80% of the total impact (ecoinvent v3.8). Despite the fact that the impact scores of the recycled blend of scenario 1 are higher than PP for most of the impact categories analysed, results are in the same order of magnitude, and the impact in these categories is expected to decrease when using full-scale industrial extruders with yield rates greater than 100 kg/h.

In the case of Scenario 2, the blend with TiO_2_ shows better performance in almost all the impact categories analysed in comparison with virgin PA66, especially low in terms of ADP fossil and ADP elements, and nearly half reduction in GWP100. Here the same reflection can be made on the use of industrial extruders, since extrusion processes are the ones that contribute most to almost all the impact categories analysed. In view of these results and from the environmental perspective, the blend with TiO_2_ (scenario 2) could be considered as a good alternative to replace virgin PA66, assuming that the characteristics and properties are adjusted to the requirements of the final application. As can be seen in Table 6, the environmental impact of PA66 is higher for all impact categories analysed in comparison with PP, scenario 1 and scenario 2. It is because PA66 is obtained from two monomers, adipic acid and hexametilendiamine (HMDA). The production stages of adipic acid and HMDA represent 44 and 49%, respectively, of the energy demand to produce PA66, and they contribute more than 60% of the total impact in all environmental impacts analysed [4].

## 4. Discussion: The Circular Economy Barriers and Challenges

Recycling is not the only element of the CE, but especially in the case of plastics, it will play an important role in the search for sustainable development, since it allows addressing multiple challenges that equally affect the economic, environmental and social dimensions. Recently, several authors have raised the barriers associated with the circular economy implementation and specifically for the plastic sector [26,27,28,29], due to the different types of plastics used and the quality and purity of the recycled secondary raw materials, some industrial branches demand high standards of raw material quality; hence, the acceptance of recycled plastic is rather low. However, other barriers must be taken into account: (i) economic, due to non-competitive prices of recycled polymers, oil price fluctuation and cost of waste management; (ii) legislative, the current regulatory framework does not yet adequately support the use of secondary plastics; (iii) social, manufactures are also sceptical about using resources coming from waste [30].

This study reports on the effort made at the pilot scale demonstration to propose two treatment routes for a specific type of plastic waste stream in order to upgrade the recycled material and generate secondary raw material that can be competitive, in terms of quality, with virgin materials available in the market. The environmental performance analysis indicates that it is necessary to use more resources (e.g., energy) in the material upcycling which represents a significant environmental impact of the recycling processes, despite that, the overall performance is better than the references virgin materials, especially for the scenario 2 (blend with TiO_2_). However, the analysis must take into account other elements of the aforementioned barriers. In economics terms, the general outlook is not promising since the market cost of the virgin materials is 0.8 and 2 euros/kg for PP and PA66, respectively; while the cost of the energy consumption for both recycling scenarios is approximately 0.6 and 0.9 euros/kg (estimate made only for stages carried out in Spain, the recycling stages are not taken into account). It denotes the necessary consumption of resources that must be allocated to improve the quality of recycled plastics and highlights the difficulty of competing economically with the virgin products derived from the oil industry.

In addition to the market cost, environmental impacts can be externalized using the values from Bruyn et al. (2018) [31,32]. The methodology proposes a representative factor for each midpoint impact category that transforms the impact as such into an economic cost [33]. The comparison of the weighting of the environmental prices for the midpoint indicators (Recipe 2013 Hierarchist LCIA method) for the two scenarios analyzed and the virgin materials are collected in Table 7 and Table 8. As can be seen, substituting the virgin materials PP and PA66 with the recycled materials from scenario 1 and scenario 2 results in an environmental price reduction of up to 2.5 times.

This also emphasizes the need for well-designed regulations to overcome barriers to the CE, the market cost of virgin plastics does not reflect the real cost of the product, internalizing the cost of all environmental externalities as seen in Table 7 throughout the plastic supply chain would allow plastic recycling processes to be more competitive with more attractive business models that would allow for a rapid implementation of CE strategies.

Making recycled plastic suitable for technical applications needs to go beyond treatment by developing high value-added, long-lasting applications that justify its high treatment cost and would better fit CE principles than the down-cycling approach. The project currently focuses its efforts on finding a method to enhance the r-O-PET quality through a reactive extrusion process. This improved r-O-PET could then be used to formulate high-value-added recycled plastics. Applications such as 3D printing will be explored, the use of polyolefins in 3D printing remains limited and the enhanced r-O-PET could be used as reinforcement for recycled polyolefins in order to improve their performance. Other applications such as thermal insulation will be looked into, by testing the suitability of the improved r-O-PET for physical foaming processes.

It is worth noting that this is an analysis carried out within a European project on a pilot plant scale. If it is scaled to an industrial level, the environmental impacts would be even lower than those obtained in this study, it can be noted that:

– The r-O-PET homogenization stage: this first extrusion can be eliminated since the industrial devices allow the feeding of flakes without clogging problems, which in this study was necessary given the dimensions. This would make subsequent recrystallization (prior to the preparation of the mixtures) not required.
– On an industrial scale, the energy efficiency of the production per kg of material and consequently the electrical consumption would be much lower.
– The recycled material would come from nearby areas, which would reduce the impact on transportation.

## 5. Conclusions

This study evaluates two routes for treating and upcycling the O-PET waste stream, which creates problems when recycled together with transparent PET in order to produce secondary raw materials that can be used for value-added applications following the circular economy approach. Two scenarios were proposed based on the composition of the recycled blends. The treatments included the processes from raw materials (recycling stages of opaque PET) to plastic pellets and were conducted experimentally at a pilot scale. Both recycled blends were evaluated using the LCA methodology with a scope from cradle to gate and considered two virgin raw materials (plastic) as reference materials when comparing the environmental performance of the proposed treatments.

The results indicate that the environmental performance of the two mixtures was slightly different, although the treatments were similar with an additional extrusion process in Scenario 2. Higher scores were reported for most of the impact categories for the blend with TiO_2_ mainly due to the energy consumption in the extrusion process. In terms of the comparison with virgin materials, the results indicated that PP reported better performance than the plastic mixture without TiO_2_, while PA66 reported high scores for all impact categories compared to the mixture with TiO_2_. However, it is worth mentioning that both treatments were carried out on a pilot scale, which provides a significant margin for improvement when using high-performance large-scale industrial extruders. In CE terms, it is confirmed that upcycling deserves a significant number of resources to improve the quality of secondary raw materials. In this sense and from the economic point of view, the market cost of raw materials is significantly low, compared to the cost of treating plastic blends and raises the need to address the challenges associated with the application of the CE.

The externality cost associated with the impact of raw materials appears as one of the elements to be considered through a well-designed regulation, since internalizing the environmental externality costs of the products derived from the oil industry would allow the production of secondary raw materials to be more competitive, and would promote the interest of developing new business models for recyclers and end users of these materials.

## Figures and Tables

**Table 1:** Physical, mechanical and flow properties of virgin raw materials and blends selected for the study.

Parameter	Material			
	PP	PA66	20w% r-O-PET/80% r-PP	20w% r-O-PET/76% r-PP/4% TiO_2_
Density, *ρ* (@ 23 °C) (kg·m^−3^)	902	1120	940	1006
MFI ^(a)^ (dg·min^−1^)	5	--	3.8	8
Elastic modulus, E (MPa)	1200	1400	1220	1260
Yielding stress, σ_Y_ (MPa)	28	55	24	26
Elongation at yield (e_y_) (%)	13	25	10	8
Specific modulus, E_sp_ (MPa·m^3^·kg^−1^)	1.33	1.25	1.30	1.24

**Table 2:** Transport stages included in the system boundaries from plastic recyclers to pilot scale extrusion plants.

Material	Distance (km)	Transport Type
r-O-PET flakes	600	Transport, freight, lorry>32 metric tons, EURO5
r-PP pellets	1350	Transport, freight, lorry>32 metric tons, EURO5

**Table 3:** Database data used in the LCIA calculations.

Module Name	Source	Name of Dataset
Electricity, Spain	Ecoinvent 3.6	market for electricity, medium voltage | electricity, medium voltage | Cutoff, U - ES
Water	Ecoinvent 3.6	market for tap water | tap water | Cutoff, U
Non-hazardous waste (disposed) - O-PET	Ecoinvent 3.6	treatment of waste polyethylene terephthalate, sanitary landfill | waste polyethylene terephthalate | Cutoff, U
Non-hazardous waste (recycled) -O-PET	Ecoinvent 3.6	waste polyethylene terephthalate, for recycling, sorted
Electricity, France	Ecoinvent 3.6	market for electricity, medium voltage | electricity, medium voltage | Cutoff, U - FR
Non-hazardous waste (disposed) - PP waste	Ecoinvent 3.6	treatment of waste polypropylene, sanitary landfill | waste polypropylene | Cutoff, U
Electricity, Netherlands	Ecoinvent 3.6	market for electricity, medium voltage | electricity, medium voltage | Cutoff, U - NL
Transport, Spain	Ecoinvent 3.6	transport, freight, lorry >32 metric ton, EURO5 - RER
TiO_2_	Ecoinvent 3.6	market for titanium dioxide | titanium dioxide | Cutoff, U
Material loss	Ecoinvent 3.6	treatment of waste polyethylene/polypropylene product, collection for final disposal | waste polyethylene/polypropylene product | Cutoff, U
Nitrogen	Ecoinvent 3.6	market for nitrogen, liquid | nitrogen, liquid | Cutoff, U

**Table 4:** Inventory of inputs and outputs included in the system boundaries for the production of 1 kg of pellets (functional unit) for both recycling scenarios.

Process			Scenario 1	Scenario 2
**O-PET waste recycling**	**Inputs**	O-PET waste	0.35 kg	0.35 kg
		*Auxiliary energy*	0.35 MJ	0.35 MJ
		*Auxiliary materials*		
		Water	18.06 kg	18.06 kg
	**Outputs**	*Products*		
		r-O-PET flakes	0.25 kg	0.25 kg
		*Waste*		
		Non-hazardous (disposed)	0.19 kg	0.19 kg
		Non-hazardous for recycling	0.07 kg	0.07 kg
**PP waste recycling**	**Inputs**	PP waste	0.90 kg	0.96 kg
		*Auxiliary energy*	0.96 MJ	1.05 MJ
		*Auxiliary materials*		
		Water	30.40 kg	32.08 kg
	**Outputs**	*Products*		
		r-PP pellets	0.89 kg	0.94 kg
		*Waste*		
		Non-hazardous (disposed)	0.19 kg	0.20 kg
**r-O-PET flakes transport**	**Inputs**	r-O-PET flakes	0.25 kg	0.25 kg
		Transport	0.15 t*km	0.15 t*km
	**Outputs**	r-O-PET flakes (transported)	0.25 kg	0.25 kg
**r-PP pellets transport**	**Inputs**	r-PP pellets	0.89 kg	0.94 kg
		Transport	1.20 t*km	1.27 t*km
	**Outputs**	r-PP pellets (transported)	0.89 kg	0.94 kg
**Drying of r-O-PET flakes**	**Inputs**	r-O-PET flakes (transported)	0.25 kg	0.25 kg
		*Auxiliary energy*		
		Electric energy	1.11 MJ	1.11 MJ
	**Outputs**	r-O-PET flakes (dried)	0.25 kg	0.25 kg
**Homogenisation of r-O-PET flakes**	**Inputs**	r-O-PET flakes (dried)	0.25 kg	0.25 kg
		*Auxiliary energy*		
		Electric energy	2.93 MJ	2.93 MJ
		*Auxiliary materials*		
		Cooling water	27.78 kg	27.78 kg
		Nitrogen	0.07 kg	0.07 kg
	**Outputs**	*Products*		
		r-O-PET pellets	0.22 kg	0.22 kg
		*Residues*		
		Material loss	0.03 kg	0.03 kg
**Re-crystallisation of r-O-PET pellets**	**Inputs**	r-O-PET pellets	0.22 kg	0.22 kg
		*Auxiliary energy*		
		Electric energy	0.51 MJ	0.51 MJ
	**Outputs**	r-O-PET pellets (crystallised)	0.22 kg	0.22 kg
**Extrusion of r-PP/TiO2 masterbatch**	**Inputs**	r-PP pellets (transported)	-	0.94 kg
		TiO2		0.05 kg
		*Auxiliary energy*		
		Electric energy	-	8.21 MJ
		*Auxiliary materials*		
		Cooling water	-	111.1 kg
	**Outputs**	*Products*		
		r-PP/TiO2 masterbatch	-	0.89 kg
		*Residues*		
		Material loss	-	0.05 kg
**Extrusion of final blend**	**Inputs**	r-O-PET pellets (crystallised)	0.22 kg	0.22 kg
		r-PP pellets (transported)	0.89 kg	-
		r-PP/TiO2 masterbatch	-	0.89 kg
		*Auxiliary energy*		
		Electric energy	12.6 MJ	12.6 MJ
		*Auxiliary materials*		
		Cooling water	125.0 kg	125.0 kg
	**Outputs**	*Products*		
		**r-PP/r-O-PET pellets**	**1 kg**	-
		**r-PP/r-O-PET/TiO2 pellets**		**1 kg**
		*Residues*		
		Material loss	0.11 kg	0.11 kg

**Table 5:** Life Cycle Impact Assessment Results for both recycling scenarios using the CML method.

Name	Scenario 1	Scenario 2	Units	Comparison (% of Increase of Impacts from Scenario 1 to Scenario 2)
Abiotic depletion	5.33 × 10^−6^	9.38 × 10^−6^	kg Sb eq	76%
Eutrophication	0.006	0.008	kg PO_4_^3-^ eq	40%
Global warming (GWP100a)	2.39	3.57	kg CO_2_ eq	50%
Acidification	0.01	0.03	kg SO_2_ eq	80%
Ozone layer depletion (ODP)	1.29 × 10^−7^	2.01 × 10^−7^	kg CFC-11 eq	56%
Abiotic depletion (fossil fuels)	25.73	39.34	MJ	53%

**Table 6:** Comparison between recycled blends and virgin polymers for Scenario 1 and Scenario 2.

Impact Category	Scenario 1		Scenario 2	
	r-PP/r-O-PET Blend	PP	r-PP/r-O-PET/TiO_2_ Blend	PA66
ADP fossil (MJ)	25.73	68.35	39.34	104.31
ADP elements (kg Sb eq)	5.33 × 10^−6^	1.33 × 10^−5^	9.38 × 10^−6^	6.53 × 10^−5^
GWP 100 (kg CO_2_ eq)	2.39	1.90	3.57	9.22
EP (kg PO_4_ eq.)	5.94 × 10^−3^	1.21 × 10^−3^	8.33 × 10^−3^	6.80 × 10^−3^
AP (kg SO_2_ eq.)	1.41 × 10^−2^	5.43 × 10^−3^	2.54 × 10^−2^	2.96 × 10^−2^
ODP (kg CFC11 eq.)	1.29 × 10^−7^	2.24 × 10^−8^	2.01 × 10^−7^	7.19 × 10^−9^

**Table 7:** Comparison between recycled blend (rPP/r-O-PET) and virgin PP for **Scenario 1** on environmental pricing [31,32].

Impact Category	Blend for Scenario 1	PP (Virgin)	Unit	Environmental Price per Environmental Impact Indicator	Unit	Blend for Scenario 1	PP (Virgin)
**Climate change**	2.35	1.83	kg CO_2_-eq.	€0.06	€/kg CO_2_-eq.	€0.13	€0.10
**Ozone depletion**	1.28 × 10^−7^	2.25 × 10^−8^	kg CFC-eq.	€30.40	€/kg CFC-eq.	€0.00	€0.00
**Acidification**	0.012	0.004	kg SO_2_-eq.	€7.48	€/kg SO_2_-eq.	€0.10	€0.04
**Freshwater** **eutrophication**	8.43 × 10^−4^	2.47 × 10^−4^	kg P-eq.	€1.86	€/kg P-eq.	€0.00	€0.00
**Marine eutrophication**	0.004	0.001	kg N	€3.11	€/kg N	€0.01	€0.00
**Human toxicity**	0.94	0.22	kg 1.4 DB-eq.	€0.10	€/kg 1.4 DB-eq.	€0.09	€0.02
**Photochemical oxidant formation**	7.89 × 10^−3^	6.05 × 10^−3^	kg NMVOC-eq.	€1.15	€/kg NMVOC-eq.	€0.01	€0.01
**Particulate matter formation**	4.70 × 10^−3^	1.81 × 10^−3^	kg PM10-eq.	€39.20	€/kg PM10-eq.	€0.18	€0.07
**Terrestrial** **ecotoxicity**	2.19 × 10^−4^	2.06 × 10^−5^	kg 1.4 DB-eq.	€8.69	€/kg 1.4 DB-eq.	€0.00	€0.00
**Freshwater** **ecotoxicity**	7.09 × 10^−2^	2.63 × 10^−2^	kg 1.4 DB-eq.	€0.04	€/kg 1.4 DB-eq.	€0.00	€0.00
**Marine ecotoxicity**	6.69 × 10^−2^	2.32 × 10^−2^	kg 1.4 DB-eq.	€0.01	€/kg 1.4 DB-eq.	€0.00	€0.00
**Ionizing radiation**	1.23	0.01	kg kBq U235-eq.	€0.05	€/kg kBq U235-eq.	€0.06	€0.00
**Land use**	3.42 × 10^−5^	1.54 × 10^−5^	m^2^	€0.08	€/m^2^	€0.00	€0.00
**Abiotic depletion (fossil fuels)**	25.73	68.35	MJ	€0.16	€/MJ	€4.12	€10.94
**Total weigh LCA score using environmental pr** **i** **cing**						**€4.71**	**€11.19**

**Table 8:** Comparison between recycled blend (rPP/r-O-PET/TiO_2_) and virgin PA66 for **Scenario 2** on environmental pricing [31,32].

Impact Category	Blend for Scenario 2	PA 66 (Virgin)	Unit	Environmental Price per Environmental Impact Indicator	Unit	Blend for Scenario 2	PA 66 (Virgin)
**Climate change**	3.52	9.27	kg CO_2_-eq.	€0.06	€/kg CO_2_-eq.	€0.20	€0.52
**Ozone depletion**	2.00 × 10^−7^	7.26 × 10^−9^	kg CFC-eq.	€30.40	€/kg CFC-eq.	€0.00	€0.00
**Acidification**	0.022	0.027	kg SO_2_-eq.	€7.48	€/kg SO_2_-eq.	€0.17	€0.21
**Freshwater eutrophication**	1.32 × 10^−3^	2.07 × 10^−4^	kg P-eq.	€1.86	€/kg P-eq.	€0.00	€0.00
**Marine eutrophication**	0.006	0.009	kg N	€3.11	€/kg N	€0.02	€0.03
**Human toxicity**	1.39	0.08	kg 1.4 DB-eq.	€0.10	€/kg 1.4 DB-eq.	€0.14	€0.01
**Photochemical oxidant formation**	1.25 × 10^−2^	2.83 × 10^−2^	kg NMVOC-eq.	€1.15	€/kg NMVOC-eq.	€0.01	€0.03
**Particulate matter formation**	7.88 × 10^−3^	9.49 × 10^−3^	kg PM10-eq.	€39.20	€/kg PM10-eq.	€0.31	€0.37
**Terrestrial ecotoxicity**	2.82 × 10^−4^	4.40 × 10^−5^	kg 1.4 DB-eq.	€8.69	€/kg 1.4 DB-eq.	€0.00	€0.00
**Freshwater ecotoxicity**	0.10	1.94 × 10^−2^	kg 1.4 DB-eq.	€0.04	€/kg 1.4 DB-eq.	€0.00	€0.00
**Marine ecotoxicity**	9.42 × 10^−2^	7.53 × 10^−3^	kg 1.4 DB-eq.	€0.01	€/kg 1.4 DB-eq.	€0.00	€0.00
**Ionizing radiation**	1.81	2.59 × 10^−3^	kg kBq U235-eq.	€0.05	€/kg kBq U235-eq.	€0.08	€0.00
**Land use**	6.73 × 10^−6^	0	m^2^	€0.08	€/m^2^	€0.00	€0.00
**Abiotic depletion (fossil fuels)**	39.34	104.31	MJ	€0.16	€/MJ	€6.29	€16.69
**Total weigh LCA score using environmental pricing**						**€7.24**	**€17.87**

## Data Availability

The data presented in this study are available on request from the corresponding author.
